# A Importância da Identificação dos Fenótipos na Hipertensão Arterial

**DOI:** 10.36660/abc.20250530

**Published:** 2025-10-13

**Authors:** Rui Póvoa

**Affiliations:** 1 Ambulatório de Hipertensão Resistente do Hospital do Servidor Público Estadual São Paulo SP Brasil Ambulatório de Hipertensão Resistente do Hospital do Servidor Público Estadual "FMO", São Paulo, SP – Brasil; 2 Universidade Federal de São Paulo São Paulo SP Brasil Universidade Federal de São Paulo, São Paulo, SP – Brasil

**Keywords:** Fenótipo, Hipertensão, Pressão Arterial

A avaliação dos níveis pressóricos envolve a compreensão da relação da pressão arterial (PA) com a saúde de forma global, e isto implica a correta medição, seguindo os critérios preconizados nas diretrizes, para encontrar o verdadeiro significado da medida no consultório e fora dele. Dentro deste espectro hipertensivo, com as medidas em momentos variáveis, conseguimos definir diversos fenótipos hipertensivos e a sua importância no risco cardiovascular. Estes conhecimentos foram se acumulando ao longo do tempo, e lapidados cada vez mais com a evolução do conhecimento médico e tecnológico.

A primeira medida experimental da PA foi feita no ano de 1711 por Stephen Halles utilizando uma cânula na artéria crural de um cavalo e conectando-a a um tubo de vidro que elevou o sangue a dois metros e meio acima da altura do animal.^1^ Entretanto, a hipertensão arterial (HA) só foi clinicamente valorizada em 1896 com a invenção do primeiro esfigmomanômetro de coluna de mercúrio pelo italiano Scipione Riva-Rocci, utilizando um manguito de 4,4 cm de largura.^2^

Em 1905, um cirurgião do exército russo Nikolai Sergeyevich Korotkov desenvolveu o método auscultatório da medida indireta da PA, descrevendo os ruídos auscultatórios da PA sistólica e diastólica, técnica utilizada ainda atualmente.^3^

Em 1945 os estudos em Framingham mostraram que a PA no consultório é um importante fator de risco para praticamente todas as doenças cardiovasculares, principalmente o acidente vascular cerebral, insuficiência cardíaca, infarto do miocárdio, etc.^4^ Foi o momento em que foi valorizada como um dos mais importantes fatores de risco cardiovascular.

Em vista da PA ser uma variável biológica e sofrer influências de diversos fenômenos internos e externos surgiu a dúvida se a simples medida na consulta médica tinha uma representatividade semelhante às medidas durante todo o dia. A avaliação fora do consultório e no sono refletem o verdadeiro cotidiano pressórico. Na década de 1960, Herbert Kain, Maurice Sokolow e Allen Hinman, desenvolveram equipamentos para medições contínuas, e não só restritas ao ambiente médico.^5^ A monitorização ambulatorial da pressão arterial (MAPA) é um método que registra a PA durante 24 horas, enquanto o paciente realiza suas atividades diárias, permitindo uma avaliação mais precisa das variações da PA, incluindo as medidas durante o sono. A medida residencial da pressão arterial (MRPA) é outra opção para a medida fora do consultório com vantagens e desvantagens em relação a MAPA, porém mais factível em populações carentes.^6^ Da mesma forma que a medida de consultório é um importante fator de risco, a medida fora do consultório se correlacionou de forma ainda mais intensa com o risco cardiovascular.^7^

A medida de consultório e fora dele permitiu a definição de diversos outros fenótipos da HA, dependendo do uso ou não de fármacos anti-hipertensivos, com características próprias e riscos peculiares. Com a evolução do conhecimento sobre a HA ao longo de mais de cem anos se verificou que a simples medida, que ainda é uma rotina na prática médica, não explicava o amplo espectro da doença hipertensiva. A avaliação fora do consultório complementa algumas lacunas no imenso complexo multifatorial da hipertensão. Com a descrição e definição destes novos conceitos sobre os diversos fenótipos, podemos entender e quantificar o risco cardiovascular da hipertensão do avental, do efeito do avental branco, da hipertensão mascarada e tantos outros fenótipos. Entretanto a prevalência e a distribuição em populações especificas ainda não é totalmente conhecida e motivo de pesquisas epidemiológicas, principalmente no Brasil.

O estudo de Barbosa et al., avaliou quatro fenótipos na população brasileira e pela primeira vez a quantificação pelo sexo. Os resultados foram interessantes pois as mulheres apresentam um melhor controle da HA. Tinham uma maior expressividade da hipertensão controlada e hipertensão do avental branco não controlada, em relação aos homens. O sexo masculino apresentou maior frequência de hipertensão sustentada não controlada e hipertensão mascarada não controlada.^8^ O método utilizado no estudo foi a MRPA que diagnostica de forma similar a MAPA os diversos fenótipos da hipertensão.^9^ Ambos os métodos apresentam valor similar em prever eventos cardiovasculares, principalmente na hipertensão mascarada e na hipertensão mascarada não controlada.^10^

Neste estudo de Barbosa et al.,^8^ o controle pressórico, no consultório e domiciliar, foi de 40,3%. Números que levantam uma bandeira vermelha e refletem a realidade brasileira. Este alerta que o estudo expõe pode servir para uma conscientização maior dos sistemas de saúde, e da necessidade de mais campanhas na população, da importância do tratamento correto e controle desta doença crônica de alta prevalência. Além disso, a identificação destes fenótipos ajuda na abordagem terapêutica mais individualizada maximizando a ação para atingir as metas e minimizando do risco ao paciente.

O conhecimento da HA evoluiu bastante desde a primeira medida de Riva-Rocci, e no caminho desta estrada os conceitos se aprimoraram ainda mais com a medicina baseada em evidências e a precisão das medidas com a incorporação tecnológica. Felizmente não existe o ponto final nesta estrada e a página da história da hipertensão é apenas um simples rabisco com um mar imenso de folhas em branco a serem escritas.
