# Supplementary material for: A Importância da Identificação dos Fenótipos na Hipertensão Arterial
Source: Arq Bras Cardiol. 2025 Oct 13;122(9):e20250530. [Article in Portuguese] doi: 10.36660/abc.20250530 (PMC12674841; doi:10.36660/abc.20250530)
Supplement: Translation

# Translation: The Importance of Identifying Phenotypes in Arterial Hypertension

**DOI:** 10.36660/abc.20250530i

**Authors:** Rui Póvoa

**Affiliations:** 1 São Paulo SP Brazil Ambulatório de Hipertensão Resistente do Hospital do Servidor Público Estadual "FMO", São Paulo, SP – Brazil; 2 São Paulo SP Brazil Universidade Federal de São Paulo, São Paulo, SP – Brazil

**Keywords:** Phenotype, Hypertension, Arterial Pressure

Assessing blood pressure (BP) levels involves understanding the relationship between BP and overall health. This requires accurate measurement, following the criteria recommended in the guidelines, to determine the true meaning of the measurement in the office and beyond. Within this hypertensive spectrum, with measurements at varying times, we can define various hypertensive phenotypes and their significance for cardiovascular risk. This knowledge has accumulated over time and has been increasingly refined with the advancement of medical and technological knowledge.

The first experimental measurement of BP was made in 1711 by Stephen Halles using a cannula in the crural artery of a horse and connecting it to a glass tube that raised the blood two and a half meters above the height of the animal.^1^However, arterial hypertension was only clinically valued in 1896 with the invention of the first mercury column sphygmomanometer by the Italian Scipione Riva-Rocci, using a 4.4 cm wide cuff.^2^

In 1905, a Russian army surgeon, Nikolai Sergeyevich Korotkov, developed the auscultatory method of indirect BP measurement, describing the auscultatory sounds of systolic and diastolic BP, a technique still used today.^3^

In 1945, studies in Framingham showed that office BP is an important risk factor for virtually all cardiovascular diseases, particularly stroke, heart failure, myocardial infarction, etc.^4^This was the time when it was recognized as one of the most important cardiovascular risk factors.

Because BP is a biological variable and is influenced by various internal and external phenomena, the question arose whether simple measurements taken during a doctor's appointment were as representative as measurements taken throughout the day. Out-of-office and sleep measurements reflect true daily BP. In the 1960s, Herbert Kain, Maurice Sokolow, and Allen Hinman developed equipment for continuous measurements, not limited to the medical setting.^5^Ambulatory blood pressure monitoring (ABPM) is a method that records BP over 24 hours while the patient carries out daily activities, allowing for a more accurate assessment of BP variations, including measurements taken during sleep. Home blood pressure monitoring (HBPM) is another out-of-office measurement option with advantages and disadvantages compared to ABPM, but it is more feasible in underserved populations.^6^Just as office measurement is an important risk factor, out-of-office measurement correlated even more strongly with cardiovascular risk.^7^

In-office and out-of-office measurements have allowed the definition of several other phenotypes of hypertension, depending on the use or non-use of antihypertensive drugs, each with its own characteristics and unique risks. As knowledge about hypertension has evolved over more than a hundred years, it has become clear that simple measurements, still routine in medical practice, do not explain the broad spectrum of hypertensive disease. Out-of-office assessment fills some gaps in the immense multifactorial complex of hypertension. By describing and defining these new concepts about the various phenotypes, we can understand and quantify the cardiovascular risk of white coat hypertension, the white coat effect, masked hypertension, and many other phenotypes. However, the prevalence and distribution in specific populations are still not fully known and are the subject of epidemiological research, particularly in Brazil.

The study by Eibel et al. evaluated four phenotypes in the Brazilian population and, for the first time, quantified them by sex. The results were interesting because women had better hypertension control. They had a greater prevalence of controlled hypertension and uncontrolled white-coat hypertension than men. Men had a higher frequency of uncontrolled sustained hypertension and uncontrolled masked hypertension.^8^The method used in the study was HBPM, which diagnoses the various hypertension phenotypes in a similar way to ABPM.^9^Both methods have similar value in predicting cardiovascular events, especially in masked hypertension and uncontrolled masked hypertension.^10^

In this study by Eibel et al.,^8^BP control, both in the office and at home, was 40.3%. These figures raise a red flag and reflect the Brazilian reality. This warning presented by the study can serve to raise awareness within healthcare systems and the need for more public campaigns on the importance of appropriate treatment and control of this highly prevalent chronic disease. Furthermore, identifying these phenotypes helps in a more individualized therapeutic approach, maximizing action to achieve goals and minimizing patient risk.

Knowledge of hypertension has evolved significantly since Riva-Rocci's first measurement, and along the way, concepts have been further refined by evidence-based medicine and the precision of measurements through the incorporation of technology. Fortunately, there is no end to this journey, and the history of hypertension is just a simple scribble with a vast sea of blank pages yet to be written.
